# Supplementary material for: On-Site Evaluation of Constituent Content and Functionality of Perilla frutescens var. crispa Using Fluorescence Spectra
Source: Molecules. 2023 Oct 20;28(20):7199. doi: 10.3390/molecules28207199 (PMC10609569; doi:10.3390/molecules28207199)
Supplement: Supplementary file 1 [file molecules-28-07199-s001.zip › SupplementaryMaterials_proof.pdf]

Supplementary Materials for

On-Site Evaluation of Constituent Content and Functionality of *Perilla frutescens* var. crispa Using Fluorescence Spectra

Hidemichi Sano, Satoru Kawaguchi, Toshifumi Iimori, Masahiro Kuragano, Kiyotaka Tokuraku, and Koji Uwai*

Graduate School of Engineering, Muroran Institute of Technology, 27-1 Mizumoto-cho, Muroran 050-8585, Japan

e-mail address

Hidemichi Sano: 22041042@mmm.muroran-it.ac.jp

Satoru Kawaguchi: skawaguchi@mmm.muroran-it.ac.jp

Toshifumi Iimori: iimori@mmm.muroran-it.ac.jp

Masahiro Kuragano: gano@mmm.muroran-it.ac.jp

Kiyotaka Tokuraku: tokuraku@mmm.muroran-it.ac.jp

Koji Uwai: uwai@mmm.muroran-it.ac.jp

* Correspondence: uwai@mmm.muroran-it.ac.jp;

Tel.: +81-0143-46-5775

CONTENTS

1. **Perilla samples** 3
2. **Fluorescence spectrum measurement** 4
3. **Chlorophyll quantification** 6
4. **Total polyphenol quantification** 7
5. **Total flavonoid content** 8
6. **Rosmarinic acid RA) quantification by LCMS** 9
7. **2,2-Diphenyl-1-picrylhydrazyl (DPPH) radical scavenging activity** 13
8. **Ferric Reducing Antioxidant Power** 18
9. **Oxygen Radical Absorbance Capacity** 19
10. **Evaluation of amyloid-beta aggregation inhibitory activity using the automated microliter-scale high-throughput screening (MSHTS) System** 21
11. **Full wavelength R^2 contour maps for NDSI, RSI and DSI analysis of *perilla*** 27
12. **Relationship between functionality and ingredient amount** 31

References 33

1. Perilla samples

Perilla frutescens var. crispa (seedlings grown at AW Farm Chitose (Chitose, Japan)) was grown in a field at our university in 2021, and three leaves from the fourth node were harvested each week (n=40). Part of the leaves was used for fluorescence spectra and chlorophyll measurements. At the same time, the rest was extracted with 99.5% ethanol (ethanol with a volume of five times the material weight) for three weeks and stored in a refrigerator at 4 ℃ until measurements of constituents and functionality. We determined that perillaldehyde, the main component of perilla, would reach saturation after one week, so there would be no problem in extracting it for three weeks and add this figure as Figure S1.

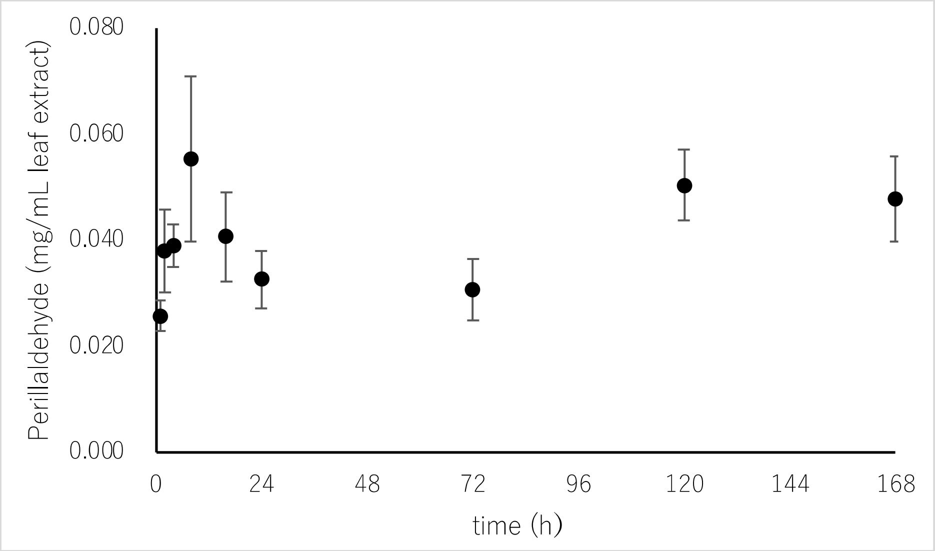

Figure S1 Perillaldehyde in perilla was measured over multiple hours.

2. Fluorescence spectrum measurement

The posterior end of the leaf was cut into 1 cm squares in Figure 9 (In tobacco leaves, it has been reported that polyphenols are present closest to the stem [1]. In addition, as shown in Figure S2, the fluorescence at 690 nm and 740 nm, the fluorescence region of chlorophyll, was measured by placing a perilla leaf on a 96-well plate, and the results were almost identical (416.0 ± 77.0, 1391.0 ± 206.8) to the average fluorescence intensity of the whole leaf (394.7 ± 113.9, 1287.3 ± 360.0), respectively (Table S2. 0, 1391.0±206.8) (Tables S1 and S2). Therefore, this position was used as the measurement position of the leaves.). The excitation light's incident angle on the cut leaves' surface was 30°. The excitation light wavelength was varied in the range 260–800 nm by 5 nm, and fluorescence spectra were measured every 0.5 nm [1]. The fluorescence spectra were corrected concerning variations in the sensitivity of the spectrofluorophotometer light detector at different wavelengths.

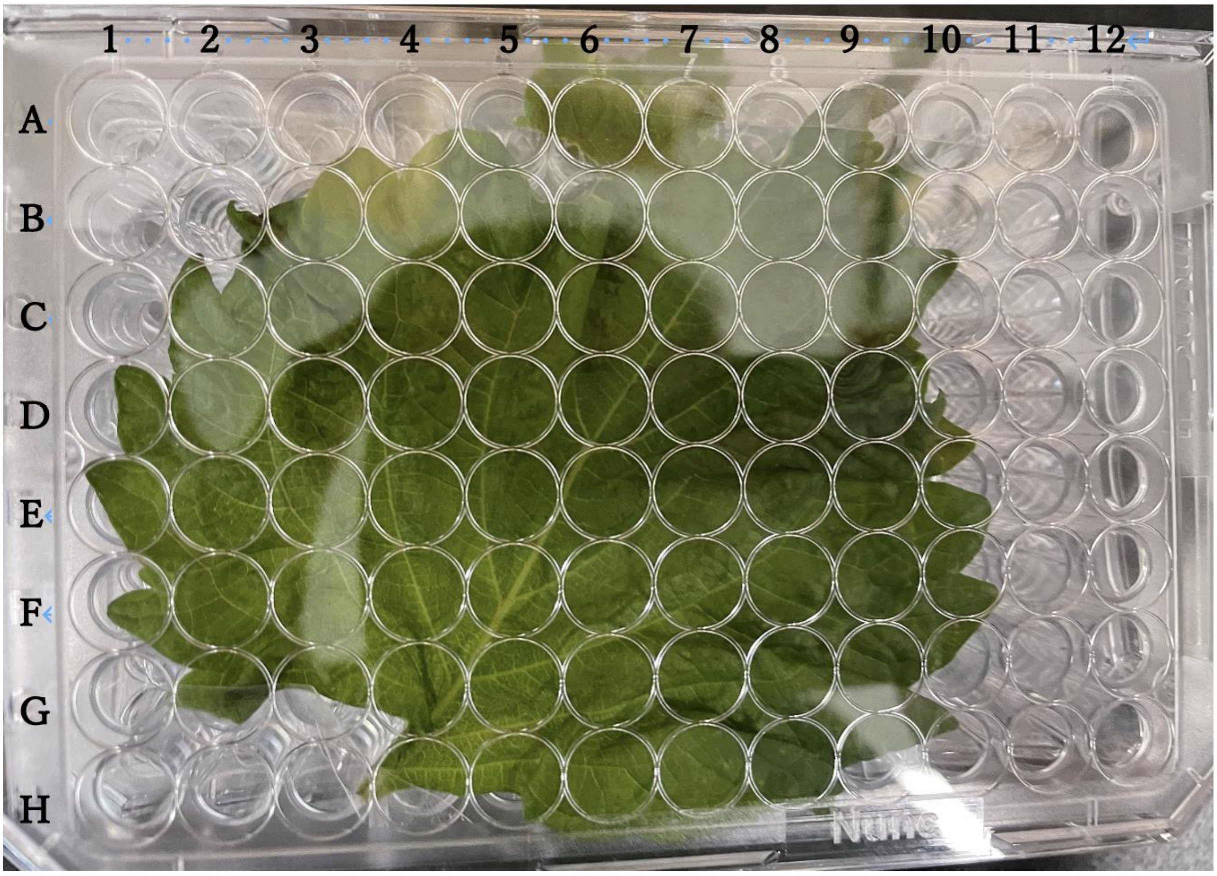

Figure S2 Diagram showing perilla leaves placed on a 96-well plate and measuring the fluorescence wavelength of the leaves

Table S1 Emission intensity of perilla leaves placed on a 96-well plate and measuring the fluorescence wavelength at 690 nm

E_m690	1	2	3	4	5	6	7	8	9	10	11	12
A	0	0	0	1	1	397	350	1	341	0	1	0
B	0	3	472	430	510	532	443	383	306	1	0	0
C	1	474	510	371	396	384	391	487	415	0	0	0
D	482	503	402	385	325	394	335	402	305	83	0	0
E	459	474	477	473	452	471	359	453	425	215	0	0
F	270	457	421	289	264	295	406	423	409	397	0	0
G	0	296	484	470	387	420	431	476	488	72	1	0
H	0	0	0	437	403	424	509	589	451	0	1	0

Table S2 Emission intensity of perilla leaves placed on a 96-well plate and measuring the fluorescence wavelength at 740 nm

E_m740	1	2	3	4	5	6	7	8	9	10	11	12
A	1	0	0	0	6	1419	1178	0	1156	0	2	1
B	1	19	1551	1355	1501	1588	1429	1298	1065	1	0	0
C	0	1453	1520	1289	1355	1222	1225	1527	1358	0	1	0
D	1611	1531	1433	1324	1097	1441	1272	1353	943	331	0	0
E	1653	1786	1585	1674	1387	1373	1036	1226	1285	693	1	0
F	1041	1511	1325	1088	926	949	1322	1367	1308	1281	0	1
G	1	835	1634	1517	1301	1407	1430	1509	1585	221	0	0
H	1	0	0	1492	1539	1346	1645	1685	1554	1	1	1

3. Chlorophyll (Chl) quantification

Chl was measured using the Porra method [2]. The leaves used in the fluorescence spectrum measurements were crushed using a homogenizer with acetone. The suspension was centrifuged for 5 min, and the supernatant was transferred to another PCR tube. This process was repeated until the green color of the precipitate completely disappeared. Ultrapure water was added to the extract to adjust the acetone concentration to 80%. The absorbance of the conditioned solution was measured at wavelengths 663.6, 646.6, and 750 nm, and the Chl concentration was calculated using Eq. (1) – Eq. (3).

$$\text{Chl } a\ (\mu g/mL) = 12.25 \times A_{663.6} - 2.85 \times A_{646.6} \quad (1)$$

$$\text{Chl } b\ (\mu g/mL) = 20.31 \times A_{646.6} - 4.91 \times A_{663.6} \quad (2)$$

$$\text{Total Chl} = \text{Chl } a + \text{Chl } b \quad (3)$$

Table S3. Chlorophyll content per mg of perilla leaves (sample harvest: 6 Aug to 19 Nov).

	Total Chrophlly			
Date	n=1	n=2	n=3	Average±SD
6-Aug	2.36			2.36±0
13-Aug	1.98			1.98±0
20-Aug	0.18			0.18±0
27-Aug	2.48			2.48±0
3-Sep	1.38	2.04	1.76	1.72±027
10-Sep	1.41	1.67	1.05	1.38±025
17-Sep	1.07	1.58	0.88	1.18±0.29
24-Sep	1.37	1.30	1.01	1.22±0.16
1-Oct	1.27	0.97	1.29	1.18±0.15
8-Oct	1.01	0.80	1.21	1.01±0.17
15-Oct	0.94	1.03	1.02	0.99±0.04
22-Oct	1.05	0.70	2.85	1.54±0.94
29-Oct	0.64	0.75	0.70	0.70±0.04
5-Nov	0.71	0.75	0.81	0.76±0.04
12-Nov	0.90	0.88	0.51	0.76±0.04
19-Nov	0.78	0.65	0.48	0.64±0.12

4. Total polyphenol content (TPC)

Each extract's total polyphenol content was determined using the Folin-Ciocalteu method [3]. Samples of perilla extract extracted with 99.5% ethanol were evaporated under the reduced pressure and dissolved in 70% methanol (1,000, 500, and 250 μg/mL). A dilute solution of 80 μL of each extract was mixed with Folin-Ciocalteu reagent (0.2 N, 400 μL) and allowed to stand at room temperature for 5 min. Sodium bicarbonate solution (7.5%, 320 μL) was added, and after 2 h of incubation at 30 °C, the absorbance at a wavelength of 760 nm was measured. A calibration curve was prepared using gallic acid as the standard, and the concentration of polyphenols was calculated as gallic acid equivalents (GAE).

Table S4. Total polyphenol content per mg of perilla leaves (equivalent to gallic acid, sample harvest: 6 Aug to 19 Nov).

		TPC		
Date	n=1	n=2	n=3	Average±SD
6-Aug	165.64			165.64±0
13-Aug	105.97			105.96±0
20-Aug	228.58			228.58±0
27-Aug	138.60			138.60±0
3-Sep	176.83	103.48	143.57	141.30±29.99
10-Sep	159.89	149.32	96.64	135.29±27.66
17-Sep	184.76	135.96	189.57	170.10±24.22
24-Sep	130.68	158.96	178.23	155.95±19.53
1-Oct	103.79	121.04	138.76	121.20±14.27
8-Oct	140.93	206.82	201.69	183.15±29.93
15-Oct	219.88	270.23	311.25	267.12±37.37
22-Oct	253.60	278.93	192.68	241.74±36.20
29-Oct	282.81	234.02	203.40	240.08±32.70
5-Nov	203.40	272.87	300.37	258.88±40.80
12-Nov	250.18	282.66	236.81	256.55±019.25
19-Nov	269.29	243.19	255.62	256.03±10.66

5. Total flavonoid content (TFC)

The total flavonoids of each extract were assessed using the $AlCl_3$ colorimetric method [4]. Samples of perilla extract extracted with 99.5% ethanol were evaporated under the reduced pressure and dissolved in methanol (1,000, 500, and 250 μg/mL). Each extract (400 μL) and $AlCl_3$ solution (2%, 400 μL) were mixed well, and after 10 min incubation at 30 ℃, the absorbance at a wavelength of 415 nm was measured. A calibration curve was prepared using quercetin as the standard, and the concentration of flavonoid was calculated as quercetin equivalents (QE).

Table S5. Total flavonoid content per mg of perilla leaves (equivalent to quercetin, sample harvest: 6 Aug to 19 Nov).

	TFC			
Date	n=1	n=2	n=3	Average±SD
6-Aug	56.92			56.92±0
13-Aug	53.55			53.55±0
20-Aug	76.25			76.25±0
27-Aug	105.80			105.80±0
3-Sep	82.14	72.17	80.54	78.28±4.37
10-Sep	96.27	87.15	64.32	82.58±13.44
17-Sep	92.31	75.97	64.36	77.55±11.46
24-Sep	60.64	78.54	54.11	64.43±10.33
1-Oct	60.92	67.33	50.59	59.61±6.90
8-Oct	61.28	65.56	104.08	76.98±19.25
15-Oct	65.44	74.97	79.50	73.30±5.86
22-Oct	50.19	47.67	38.22	45.35±5.15
29-Oct	33.61	40.46	34.33	36.13±3.07
5-Nov	40.82	31.33	32.65	34.93±4.20
12-Nov	32.77	20.84	43.70	32.44±9.34
19-Nov	28.81	35.89	23.64	29.45±5.02

6. RA quantification by LCMS.

LCMS-8045 with ESI and LC-2060C (Shimadzu, Kyoto, Japan) equipped with Kinetex® C18 (2.1 × 150 mm, 5 µm, Phenomenex) column (40 ℃) were used for LC-MS analysis. Peaks were detected at a wavelength of 254 nm, and 0.1% formic acid in 10% methanol/water (A), and 0.1% formic acid in methanol (B) were used as mobile phases. The injection volume was 5 µL, and the gradient at this time was as follows (A to B): 0 min; 100:0, 4 min; 80:20, 9 min; 75:25, 14 min; 62:38, 17 min; 50:50, 21 min; 30:70, 22min; 25:75, 35 min; 0:100, 45min; 100:0. MS conditions were as follows: nebulizer gas flow 3 L/min, heating gas flow 10 L/min, interface temperature 300 °C, DL temperature 250 °C, heat block temperature 400 °C, draining gas flow 10 L/min, precursor ion 359 m/z, product ion 161 and 197 m/z. SRM mode was used. We generated calibration curves for RA in the range of 0.1 to 1.0 µg/mL, as shown in Figure S5. Calibration curves were prepared using standards treated in the same manner as the perilla samples, and linearity was obtained with a correlation coefficient of 0.92. All measured samples fell within the 95% confidence interval of the calibration curve.

Table S6. Rosmarinic acid content per milligram of perilla leaves (µg/mg leaf, Sample harvest: 6 Aug to 19 Nov)

	RA			
Date	n=1	n=2	n=3	Average±SD
6-Aug	29.51			29.51
13-Aug	20.96			20.96
20-Aug	47.98			47.98
27-Aug	22.97			22.97
3-Sep	24.43	27.09	27.28	26.27±1.30
10-Sep	21.97	29.75	31.94	27.89±4.28
17-Sep	43.65	50.28	43.47	45.80±3.17

24-Sep	44.68	38.25	64.56	49.16±11.2
1-Oct	50.35	52.09	53.83	52.09±1.42
8-Oct	55.58	57.32	59.06	57.31±1.42
15-Oct	60.80	62.54	64.28	62.54±1.42
22-Oct	66.02	67.77	69.51	67.77±1.42
29-Oct	71.25	72.99	74.73	72.99±1.42
5-Nov	76.47	78.22	79.96	78.22±1.42
12-Nov	81.70	83.44	85.18	83.44±1.42
19-Nov	107.87	40.80	46.00	85.18±30.5

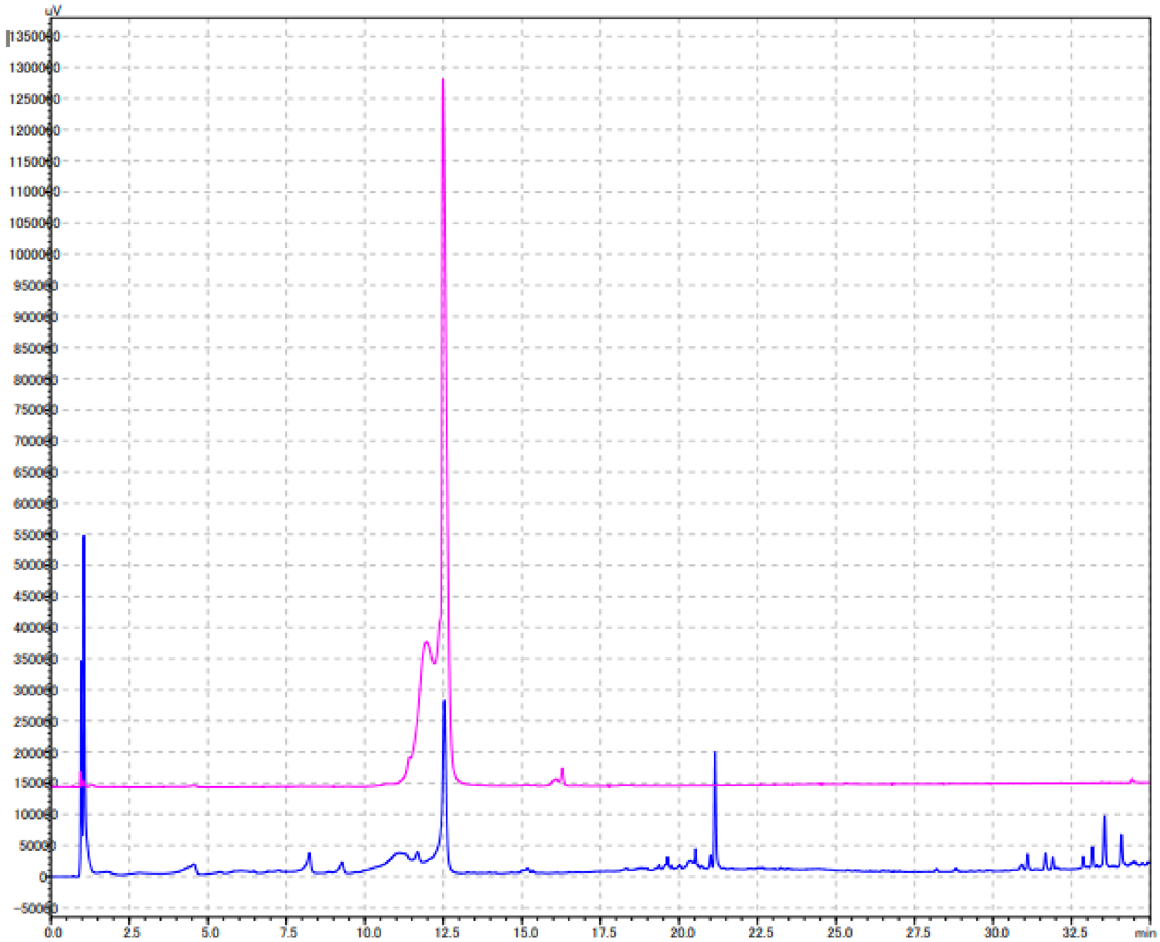

Figure S3. HPLC chromatograms of rosmarinic acid standard (1 mg/mL, top (pink)) and perilla extract (1 mg/mL, bottom (blue)) detected at 254 nm.

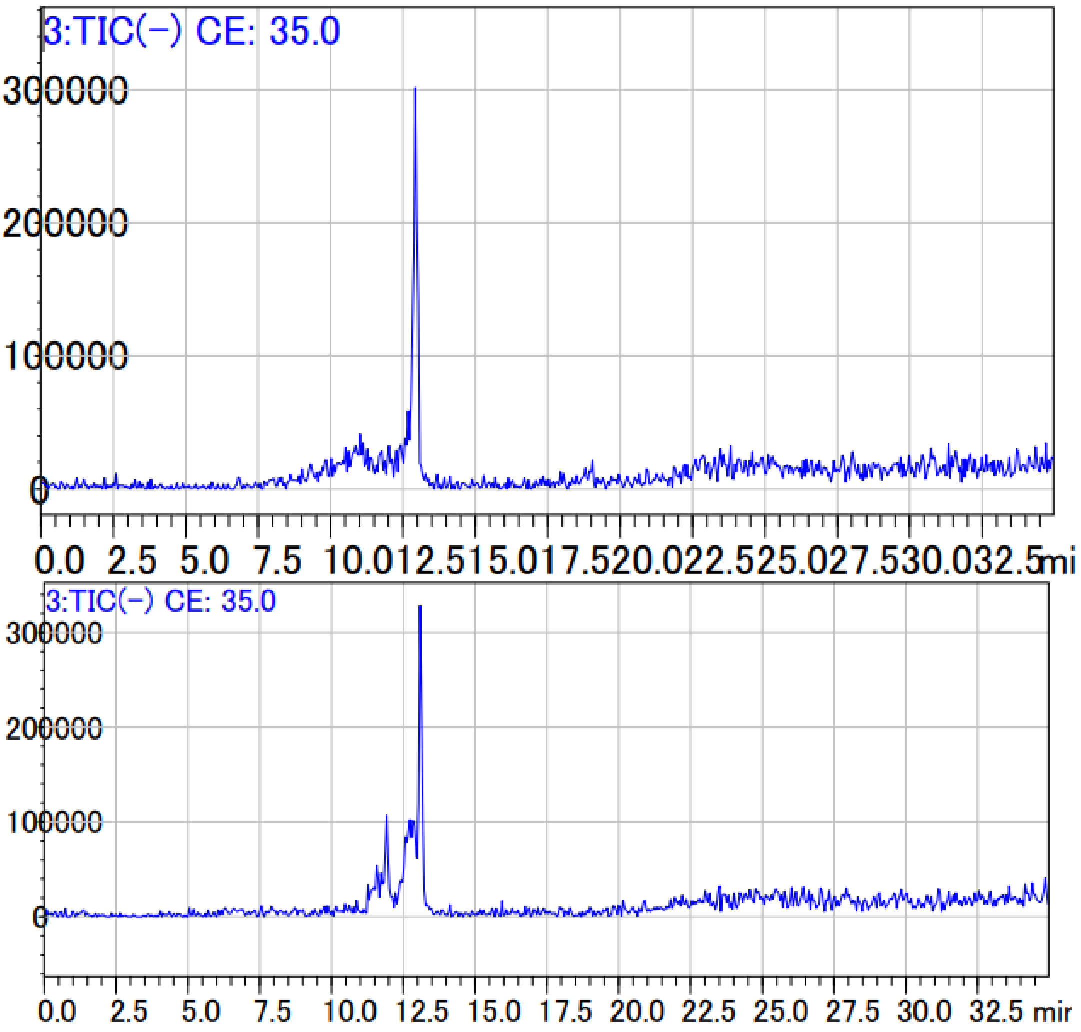

Figure S4. LC-MS chromatograms of rosmarinic acid standard (10ppm, upper) and perilla extract (0.5 ppm, lower) in TIC (TIC is the sum of precursor ion 359 m/z, product ion 161 and 197 m/z.) .

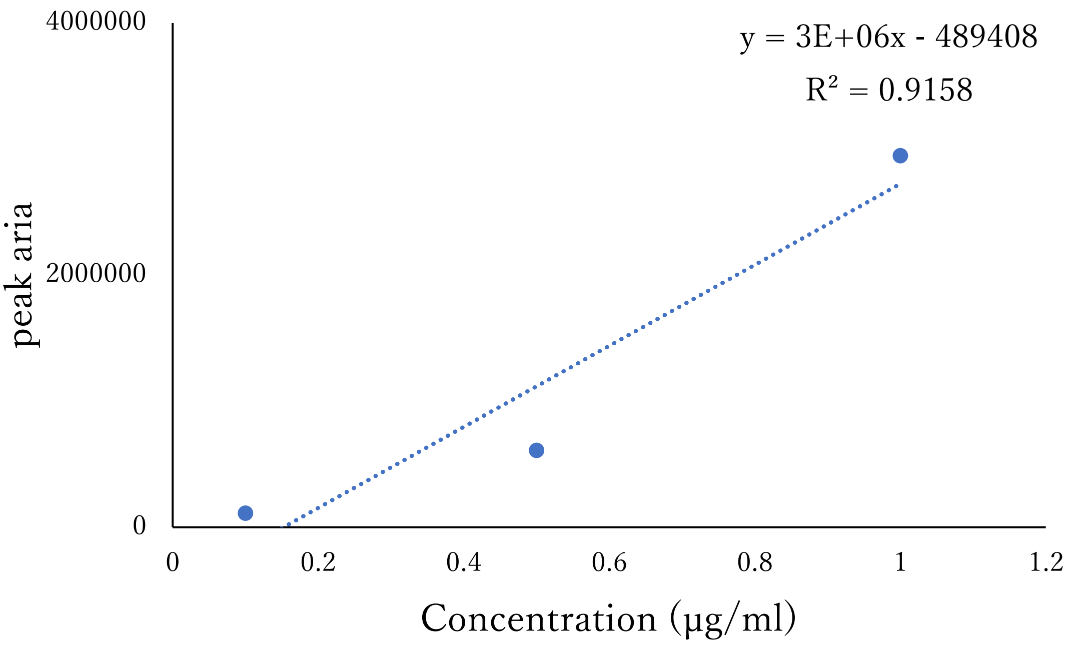

Figure S5. Rosmarinic acid calibration curve using LC-MS/MS

7. 2,2-diphenyl-1-picrylhydrazyl (DPPH) radical scavenging activity [5]

Perilla extracted with 99.5% ethanol was evaporated under reduced pressure and dissolved in ethanol (1,000, 500, 250, 125, 62.5, 31.25, 15.625, and 7.8125 μg/mL). Each sample (400 μL) was mixed with 0.01 mM DPPH in 99.5% ethanol (400 μL), and 200 μL each was transferred to a 96-well microplate reader and incubated at 30 °C for 2 h. The absorbance at a wavelength of 517 nm was measured, and the data obtained were substituted into the following equation (Eq. (4)) to calculate the inhibition rate of DPPH oxidation. The IC_{50} values were calculated from the inhibition curve by plotting the inhibition rate against the concentration of the sample.

$$\textbf{Inhibition rate}(\%) = \frac{A_{control} - A_{sample}}{A_{control}} \quad (4)$$

Table S7. DPPH radical scavenging activity of perilla leaves (IC_{50}, sample harvested 8/6 to 11/19)

	DPPH			
Date	n=1	n=2	n=3	Average±SD
6-Aug	30.82			30.82±0
13-Aug	48.42			48.42±0
20-Aug	16.31			16.31±0
27-Aug	56.01			56.01±0
3-Sep	36.49	68.07	45.88	50.15±13.24
10-Sep	139.90	72.88	23.97	78.92±47.52
17-Sep	40.77	38.50	32.15	37.14±3.65
24-Sep	39.30	51.89	28.06	39.75±9.73
1-Oct	33.98	25.30	29.86	29.71±3.55
8-Oct	45.89	47.28	48.74	47.30±1.16
15-Oct	21.32	18.88	19.34	19.85±1.06
22-Oct	36.04	31.41	24.40	30.62±4.79
29-Oct	9.86	18.33	18.09	15.43±3.94
5-Nov	15.17	11.44	0.14	8.91±6.39

12-Nov	16.18	19.82	19.18	18.39±1.59
19-Nov	19.40	21.26	25.43	22.03±2.52

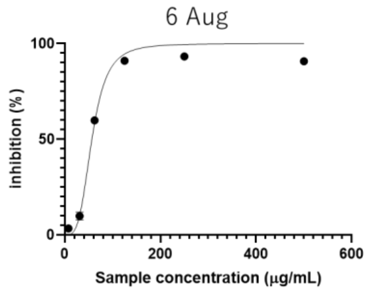

Figure S6. Inhibition curve for the DPPH radical scavenging activity of extracts of perilla leaves on 6 August.

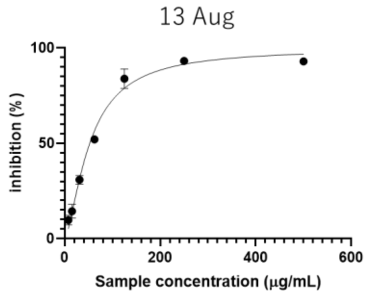

Figure S7. Inhibition curve for the DPPH radical scavenging activity of extracts of perilla leaves on 13 August.

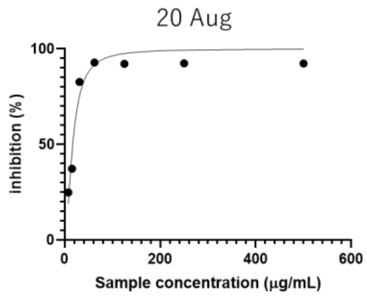

Figure S8. Inhibition curve for the DPPH radical scavenging activity of extracts of perilla leaves on 20 August.

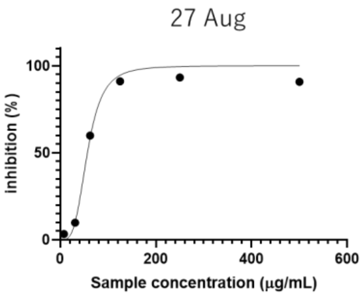

Figure S9. Inhibition curve for the DPPH radical scavenging activity of extracts of perilla leaves on 27 August.

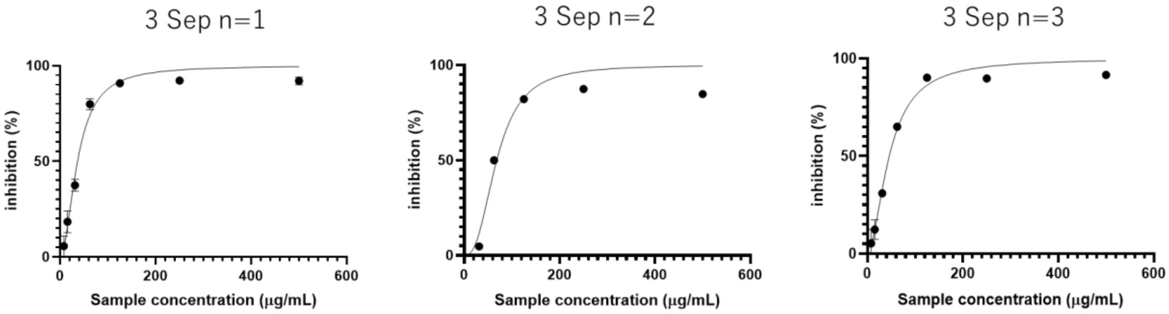

Figure S10. Inhibition curve for the DPPH radical scavenging activity of extracts of perilla leaves on 3 September.

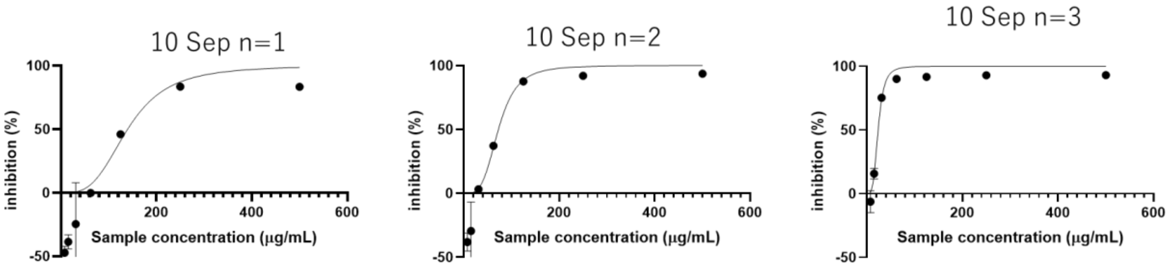

Figure S11. Inhibition curve for the DPPH radical scavenging activity of extracts of perilla leaves on 10 September.

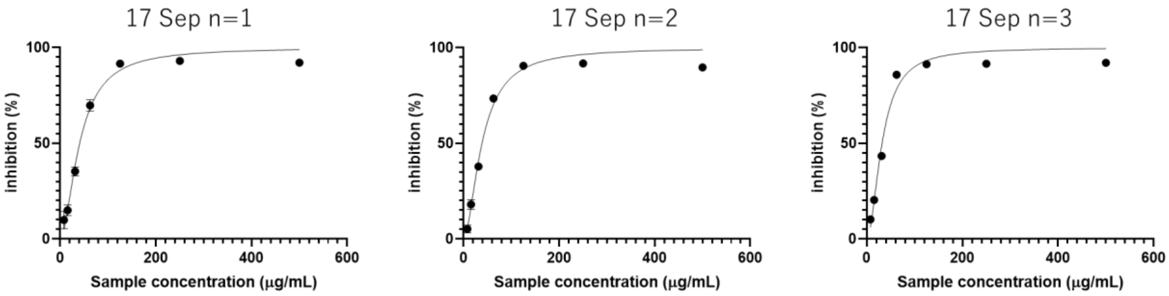

Figure S12. Inhibition curve for the DPPH radical scavenging activity of extracts of perilla leaves on 17 September.

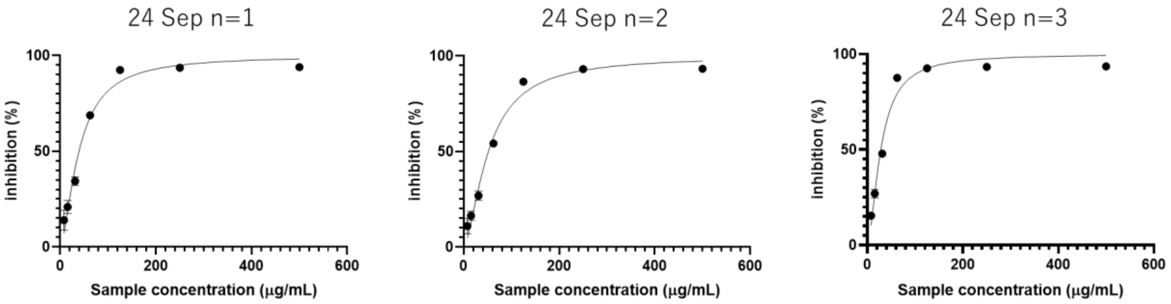

Figure S13. Inhibition curve for the DPPH radical scavenging activity of extracts of perilla leaves on 24 September.

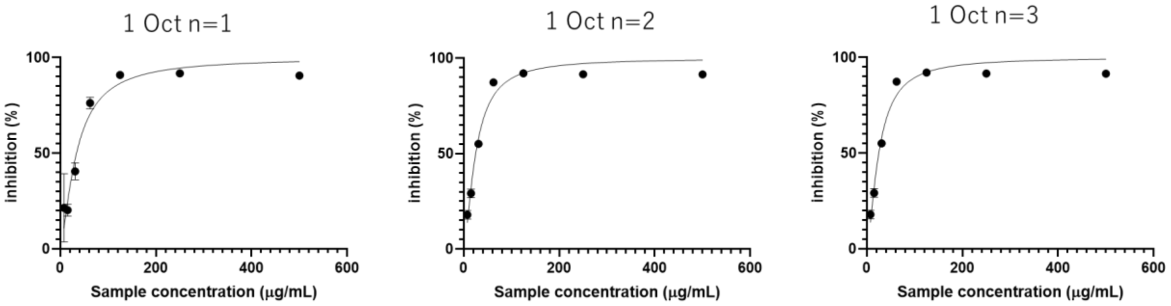

Figure S14. Inhibition curve for the DPPH radical scavenging activity of extracts of perilla leaves on 1 October.

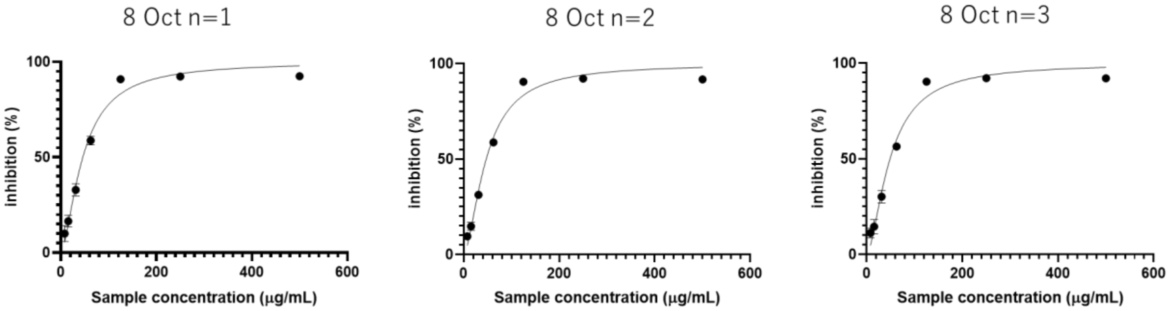

Figure S15. Inhibition curve for the DPPH radical scavenging activity of extracts of perilla leaves on 8 October.

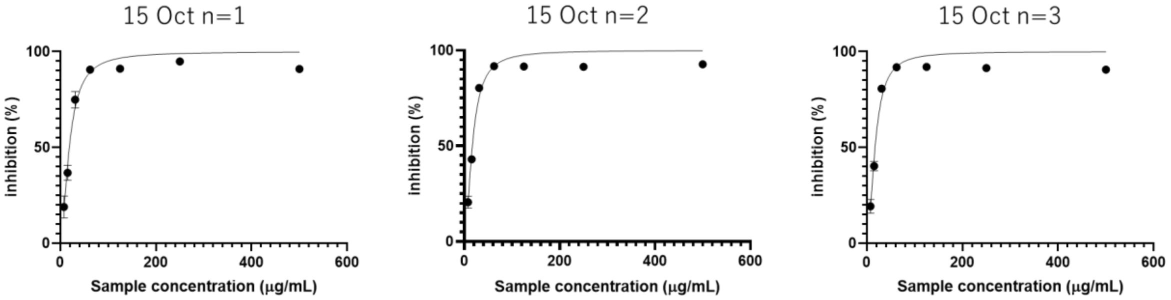

Figure S16. Inhibition curve for the DPPH radical scavenging activity of extracts of perilla leaves on 15 October.

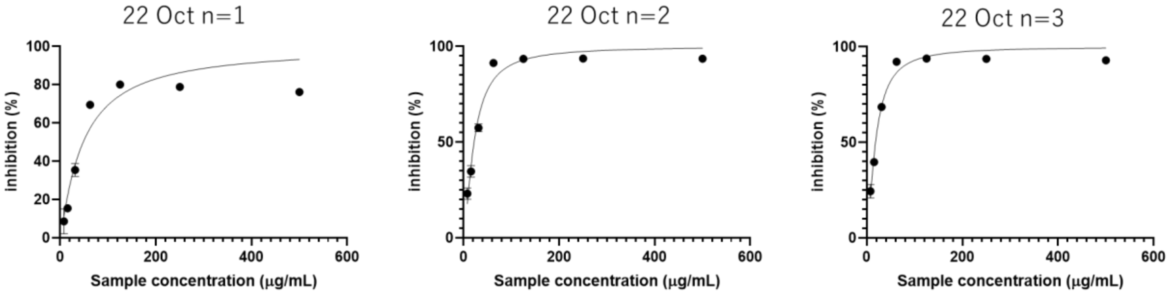

Figure S17. Inhibition curve for the DPPH radical scavenging activity of extracts of perilla leaves on 22 October.

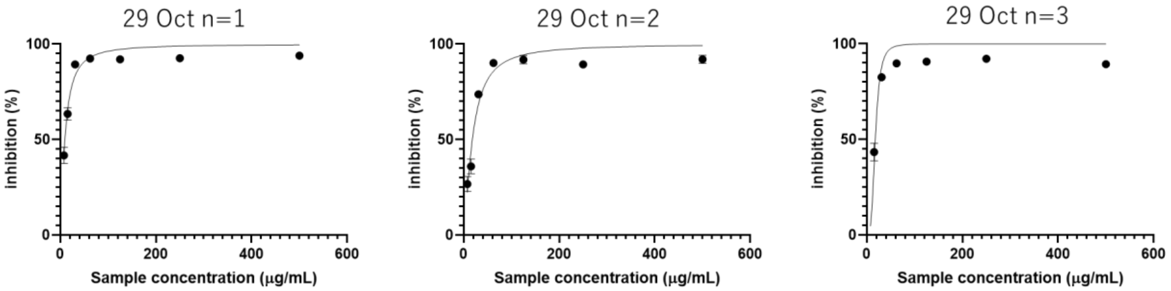

Figure S18. Inhibition curve for the DPPH radical scavenging activity of extracts of perilla leaves on 29 October.

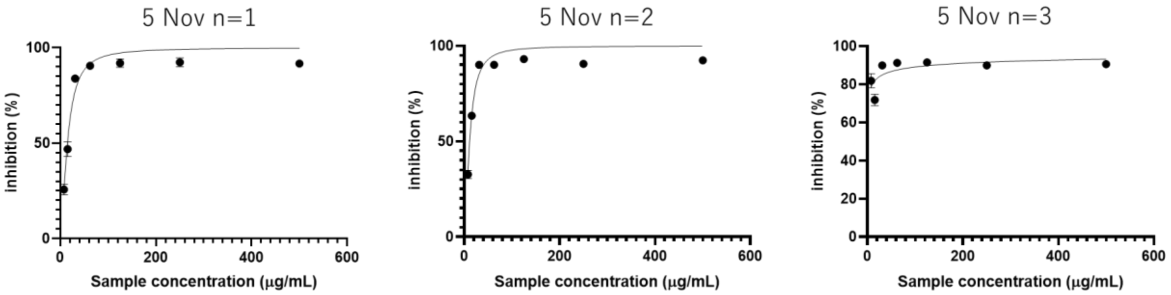

Figure S19. Inhibition curve for the DPPH radical scavenging activity of extracts of perilla leaves on 5 November.

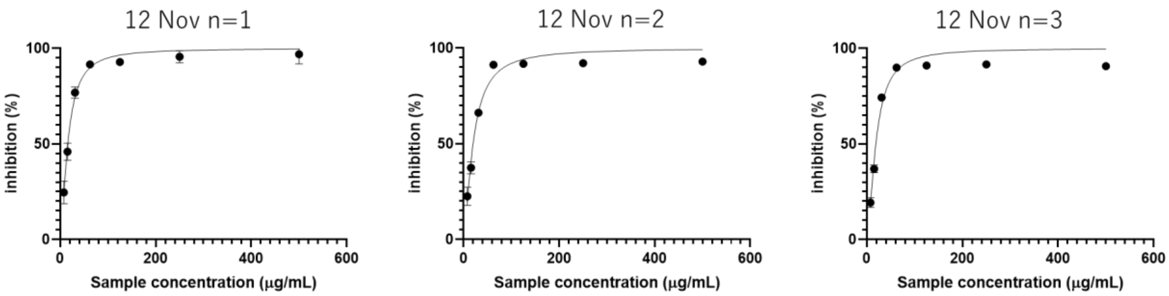

Figure S20. Inhibition curve for the DPPH radical scavenging activity of extracts of perilla leaves on 12 November.

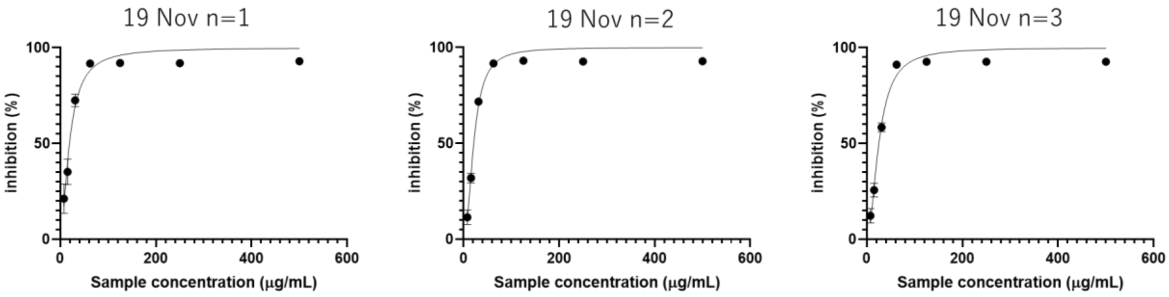

Figure S21. Inhibition curve for the DPPH radical scavenging activity of extracts of perilla leaves on 19 November.

8. Ferric reducing antioxidant power (FRAP)

FRAP was measured using the Oyaizu method [6] with some modifications[7]. Samples of perilla extract extracted with 99.5% ethanol were evaporated under reduced pressure, and dissolved in ethanol to 1 mg/mL, then diluted to 500, 250, and 125 μg/mL. To an 8 μL of each dilution, 99.5% ethanol (72 μL), ultrapure water (400 μL), 1 M HCl (120 μL), 1% $K_3[Fe(CN)_6]$ (120 μL), 1% SDS (40 μL), and 2% $FeCl_2$ (40 μL) were added. The mixture was placed in a water bath at 50 °C for 20 min, and the mixture was cooled to room temperature and mixed well. The reducing power of each extract was determined by measuring the increase in the absorbance at 750 nm. Ascorbic acid was used as the positive control and FRAP was calculated as ascorbic acid equivalents (AAE).

Table S8. FRAP of perilla leaves (ascorbic acid equivalent, sample harvest: 6 Aug to 19 Nov).

	FRAP			
Date	n=1	n=2	n=3	Average±SD
6-Aug	64.80			64.80±0
13-Aug	51.49			51.49±0
20-Aug	84.04			84.04±0
27-Aug	39.65			39.65±0
3-Sep	62.83	58.88	71.71	64.47±5.36
10-Sep	56.91	82.56	149.13	96.20±38.87
17-Sep	92.42	102.28	79.60	91.43±9.29
24-Sep	85.02	54.45	114.12	84.53±24.36
1-Oct	66.78	73.19	80.58	73.52±5.64
8-Oct	104.26	62.34	93.90	86.83±17.83
15-Oct	120.53	138.28	109.19	122.67±11.97
22-Oct	140.26	112.64	119.54	124.15±11.74
29-Oct	316.81	294.13	289.69	300.21±11.88
5-Nov	267.99	281.80	367.61	305.80±44.07
12-Nov	246.78	267.50	174.29	229.521±39.96
19-Nov	248.26	238.89	167.87	218.34±35.89

9. Oxygen radical absorbance capacity (ORAC) [8]

500 μL of the ethanol extract was evaporated under reduced pressure. The residue was dissolved in an AWA solution (acetone, ultrapure water, and acetic acid (70:29.5:0.5)) to a 12.5 μg/mL concentration. The samples were diluted with assay buffer (75 mM K_2HPO_4 solution and 75 mM KH_2PO_4 solution adjusted to pH 7.4) to 12.5, 6.25, 3.125, and 1.5625 μg/mL. To the sample (20 μL) or assay buffer (control, 20 μL) was added the fluorescein (FL) working solution (36 ng/mL of FL in assay buffer, 200 μL), placed in a 96-well microplate reader, and after shaking and stirring at 37 °C, the fluorescence intensity (E_m: 520 nm) was measured at E_x: 485 nm and, which were defined as f_{0min}. After incubating at 37 °C for 10 min, 2,2′-azobis(2-methylpropionamidine) dihydrochloride (AAPH) solution (8.6 mg/mL of AAPH in assay buffer, 75 μL) was added, and E_m: 520 nm was measured every 2 min for 90 min (f_{2min} to f_{90min}). The standard solutions were 12.5, 6.25, 3.125, and 1.5625 μg/mL samples (20 μL) of Trolox.

$$AUC = 2\ (0.5\times f_{8min} + f_{10min} + f_{12min} + f_{14min} + \ldots + f_{88min} + 0.5\times f_{90min}) / f_{0min} \quad (5)$$

$$netAUC_{Trolox} = AUC_{Trolox} + AUC_{Blank} \quad (6)$$

$$netAUC_{sample} = AUC_{sample} + AUC_{Blank} \quad (7)$$

A quadratic regression equation ($y=ax^2+bx+c$) was calculated using the concentration of each Trolox solution on the x-axis and the net AUC_{Trolox} of each Trolox solution on the y-axis.

From the regression equation, the ORAC value was calculated from Eq. (8).

$$ORAC\left(\frac{\mu mol\ TE}{g}\right) = \frac{\left(a \times \left(netAUC_{sample}\right)^2 + b \times \left(netAUC_{sample}\right) + c\right) \times V \times D}{W} \quad (8)$$

TE: Trolox equivalents; a, b, c: a, b, c in the quadratic regression equation; V: volume of sample stock solution; D: dilution factor of sample stock solution; W: sample weight.

Table S9. ORAC of perilla leaves (Trolox equivalent, sample harvest: 6 Aug to 19 Nov).

	ORAC			
Date	n=1	n=2	n=3	Average±SD
6-Aug	20.88			20.88±0
13-Aug	11.77			11.77±0
20-Aug	34.89			34.89±0
27-Aug	11.35			11.35±0
3-Sep	11.99	4.77	8.52	8.43±2.95
10-Sep	9.57	18.26	12.24	13.36±3.63
17-Sep	15.22	13.33	21.79	16.78±3.63
24-Sep	14.78	13.24	19.34	15.79±2.59
1-Oct	4.05	1.41	0.33	1.93±1.56
8-Oct	24.35	6.48	8.35	13.06±8.02
15-Oct	12.39	56.90	45.49	38.26±18.88
22-Oct	29.93	64.54	13.94	36.13±21.12
29-Oct	14.91	19.72	22.02	18.88±2.96
5-Nov	25.97	26.75	21.59	24.77±2.27
12-Nov	1.43	35.22	25.70	20.78±14.22
19-Nov	37.92	32.39	41.26	37.19±3.66

10. Evaluation of amyloid-beta aggregation inhibitory activity using the automated microliter-scale high-throughput screening (MSHTS) system [9, 10]

For MSHTS, human $A\beta_{42}$ was purchased from a commercial source (4349-v; Peptide Institute Inc., Osaka, Japan). The quantum-dot (QD) Aβ nanoprobe was prepared using QD-PEG-NH_2 (QdotTM 605 ITK^{TM} Amino (PEG) Quantum dot; Q21501MP, Waltham, MA, USA, Thermo Fisher Scientific) and Cys-conjugated $A\beta_{40}$ (23519, Anaspec Inc., Fremont, CA, USA). The half maximal effective concentration (EC_{50}) values of all plant extracts were determined by a modified automated MSHTS system. Specifically, mixed solutions were prepared with six concentrations of extracts, 25 nM QDAβ, and 25 μM $A\beta_{42}$ in PBS containing 5% ethanol and 2.5% dimethyl sulfoxide and incubated in a 1536-well plate (782096, Greiner, Kremsmünster, Austria) at 37 °C for 24 h. Images of each well were captured before and after incubation using an inverted fluorescence microscope (ECLIPSE Ti-E; Nikon) equipped with a color CMOS camera (DS-Ri2; Nikon). QD fluorescence was imaged using a 4× objective lens (Plan Apo λ 4×/0.2, Nikon) and a TRITC filter set (TRITC-A-Basic-NTE, Semrock, NY, USA). The standard deviation (SD) values of the central images of the region of interest (432 × 432 pixels) in each well were measured using the General Analysis program NIS-Elements (Nikon). Images with the highest extract concentrations, including insoluble substances, were eliminated because they affected the SD values and disrupted accurate evaluation. The EC_{50} was estimated from the SD values using Prism software (GraphPad Software, San Diego, CA, USA) with an EC_{50} shift by global fitting (asymmetric sigmoidal, five-parameter logistic)

Table S10. Aβ aggregation inhibitory activity of perilla leaves (sample harvest: 6 Aug to 19 Nov).

	Aβ aggregation inhibitory activity			
Date	n=1	n=2	n=3	Average±SD
6-Aug	0.058			0.058±0
13-Aug	0.045			0.045±0
20-Aug	0.022			0.022±0
27-Aug	0.034			0.034±0
3-Sep	0.031	0.065	0.048	0.048±0.014
10-Sep	0.093	0.029	0.030	0.051±0.030
17-Sep	0.041	0.056	0.038	0.045±0.0082
24-Sep	0.045	0.053	0.025	0.041±0.012
1-Oct	0.024	0.021	0.020	0.022±0.0014
8-Oct	0.040	0.023	0.033	0.032±0.0069
15-Oct	0.011	0.023	0.022	0.019±0.0055
22-Oct	0.026	0.040	0.023	0.030±0.0077
29-Oct	0.015	0.022	0.007	0.014±0.0062
5-Nov	0.067	0.021	0.021	0.036±0.022
12-Nov	0.018	0.024	0.023	0.021±0.0023
19-Nov	0.016	0.029	0.044	0.03±0.011

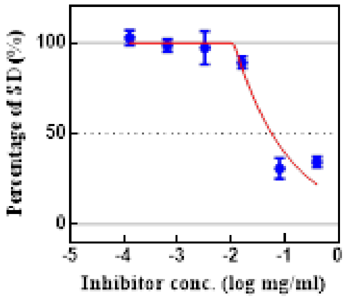

Figure S22. Inhibition curve for the Aβ aggregation inhibitory activity of extracts of perilla leaves on 6 August.

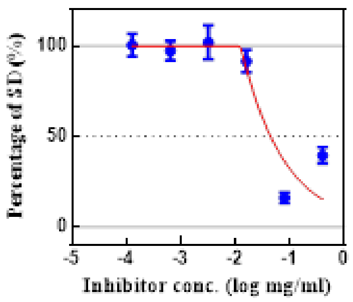

Figure S23. Inhibition curve for the Aβ aggregation inhibitory activity of extracts of perilla leaves on 13 August.

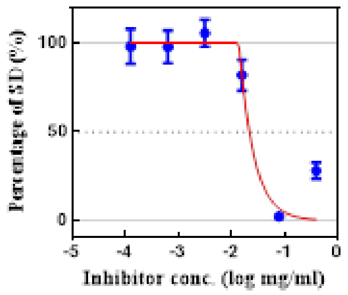

Figure. S24. Inhibition curve for the Aβ aggregation inhibitory activity of extracts of perilla leaves on 20 August.

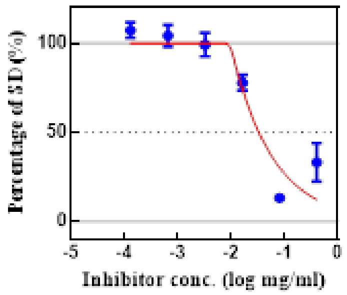

Figure S25. Inhibition curve for the Aβ aggregation inhibitory activity of extracts of perilla leaves on 27 August.

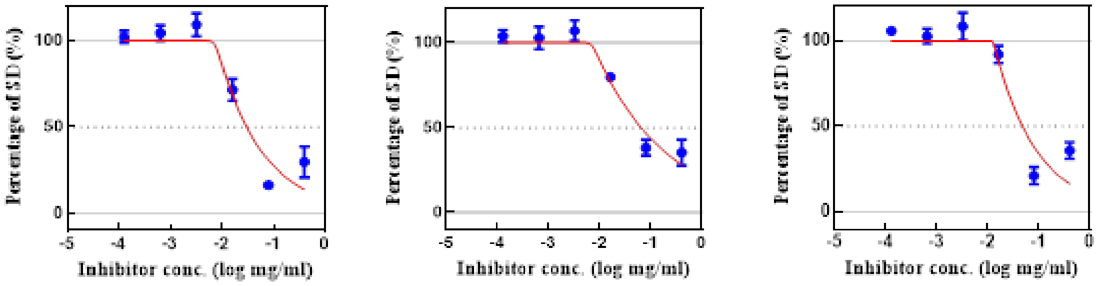

Figure S26. Inhibition curve for the Aβ aggregation inhibitory activity of extracts of perilla leaves on 3 September.

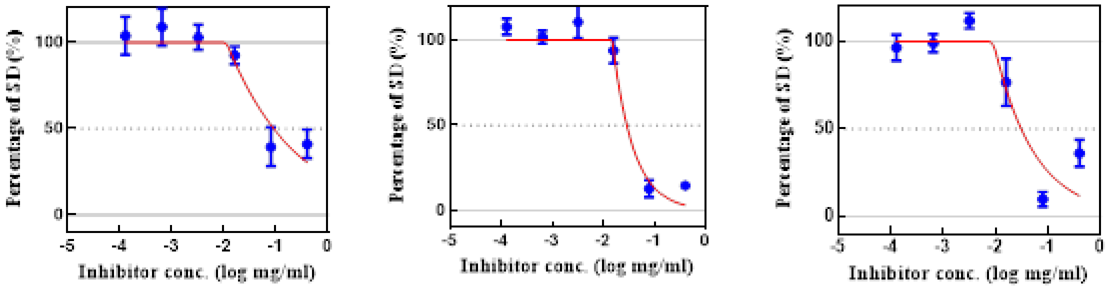

Figure S27. Inhibition curve for the Aβ aggregation inhibitory activity of extracts of perilla leaves on 10 September.

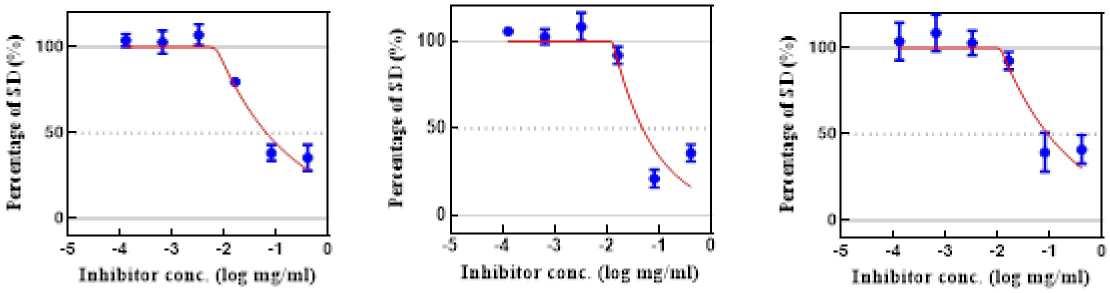

Figure S28. Inhibition curve for the Aβ aggregation inhibitory activity of extracts of perilla leaves on 17 September.

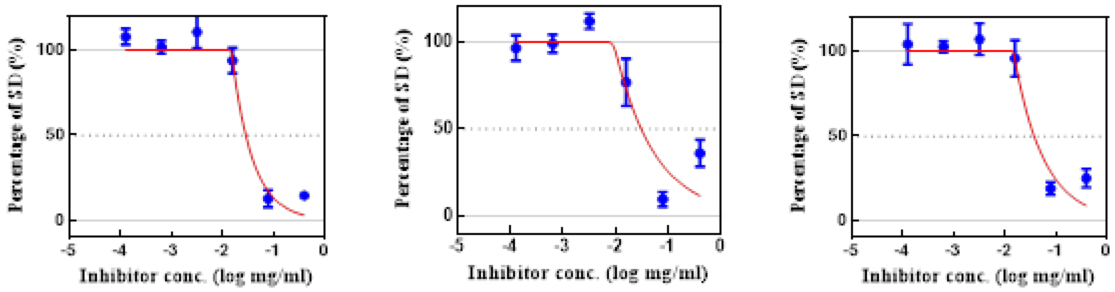

Figure S29. Inhibition curve for the Aβ aggregation inhibitory activity of extracts of perilla leaves on 24 September.

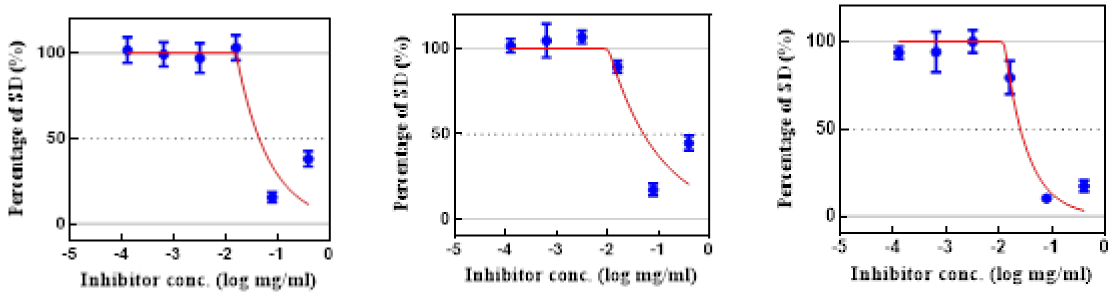

Figure S30. Inhibition curve for the Aβ aggregation inhibitory activity of extracts of perilla leaves on 1 October.

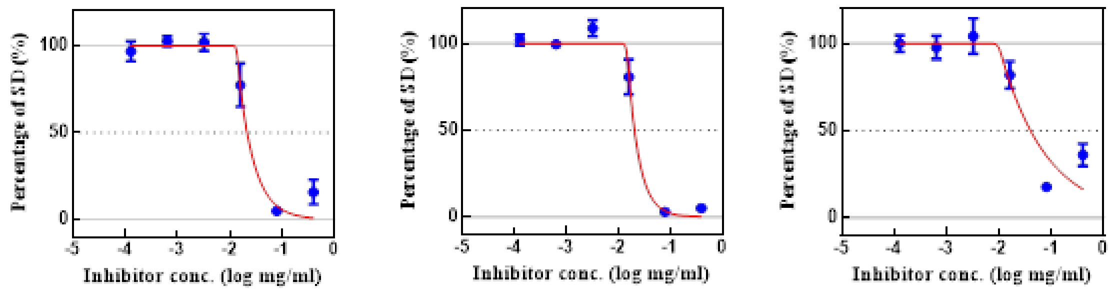

Figure S31. Inhibition curve for the Aβ aggregation inhibitory activity of extracts of perilla leaves on 8 October

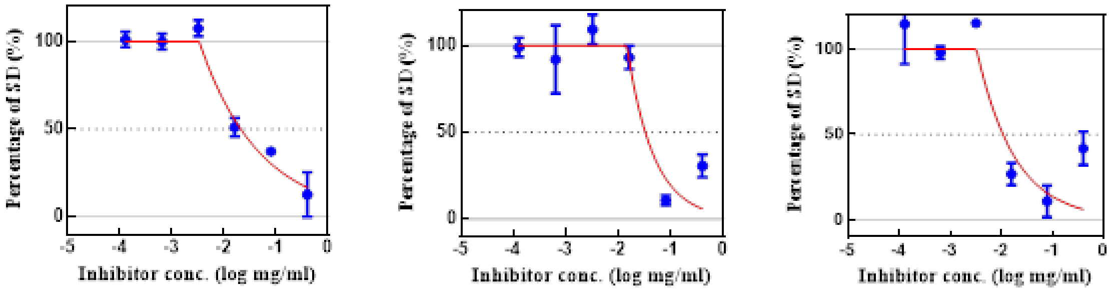

Figure S32. Inhibition curve for the Aβ aggregation inhibitory activity of extracts of perilla leaves on 15 October.

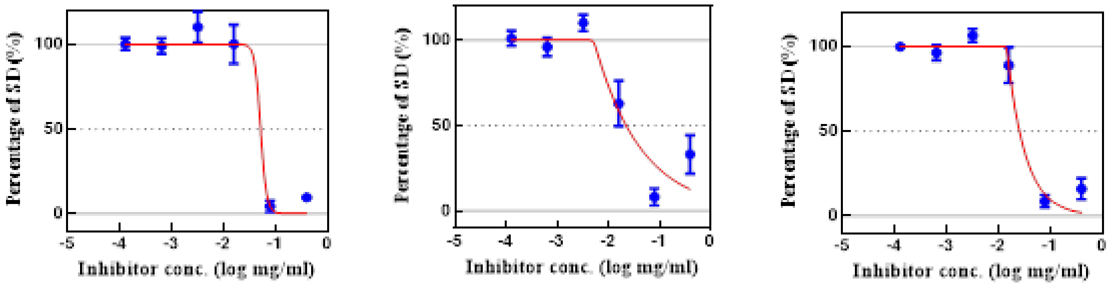

Figure S33. Inhibition curve for the Aβ aggregation inhibitory activity of extracts of perilla leaves on 22 October.

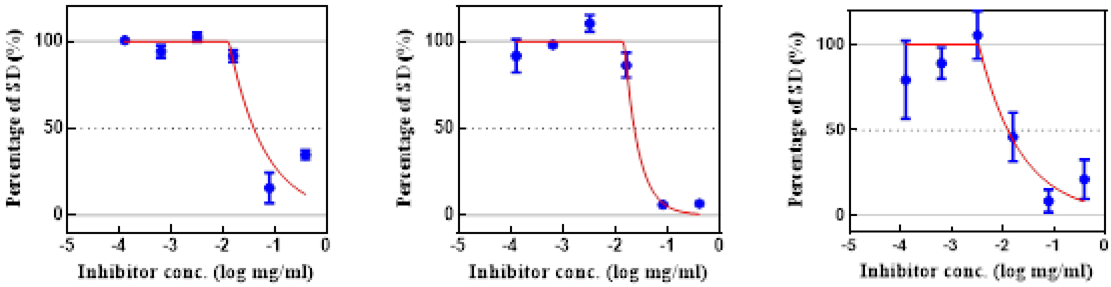

Figure S34. Inhibition curve for the Aβ aggregation inhibitory activity of extracts of perilla leaves on 29 October.

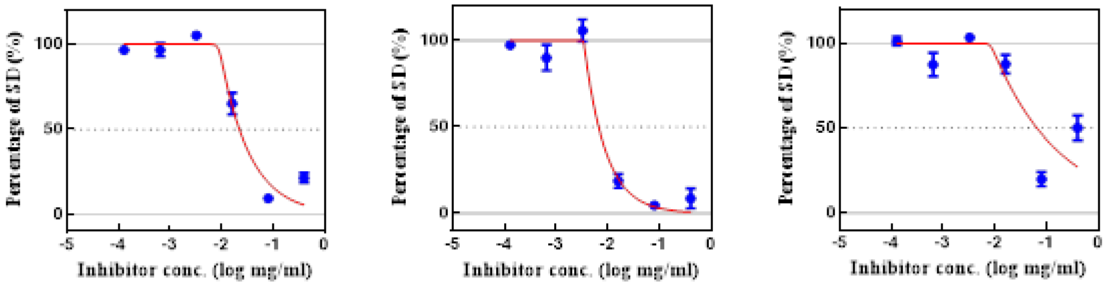

Figure S35. Inhibition curve for the Aβ aggregation inhibitory activity of extracts of perilla leaves on 5 November.

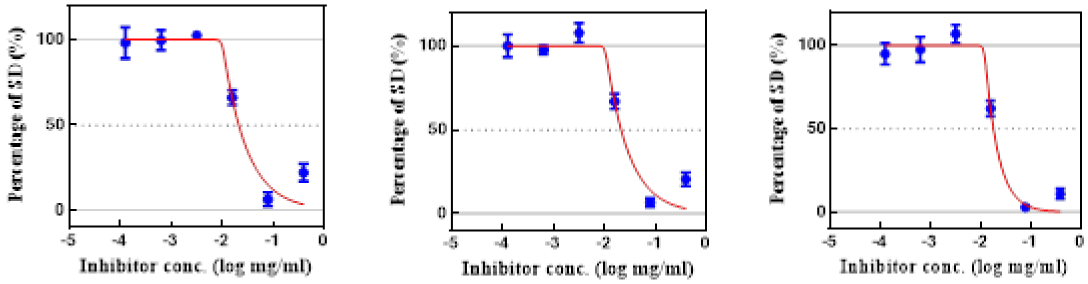

Figure S36. Inhibition curve for the Aβ aggregation inhibitory activity of extracts of perilla leaves on 12 November.

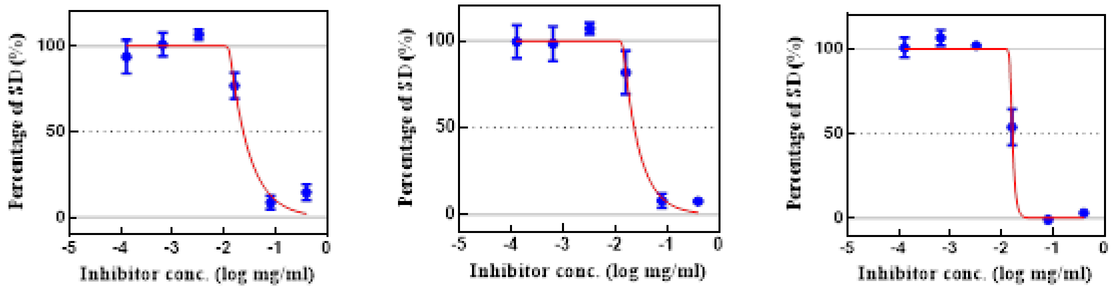

Figure S37. Inhibition curve for the Aβ aggregation inhibitory activity of extracts of perilla leaves on 19 November.

11. Full wavelength R^2 contour maps for NDSI, RSI and DSI analysis of perilla

Contour maps were produced for each excitation light irradiation ranging from 250 to 600 nm × 5 nm wavelength range. Figure. S35. to S58 in Supplementary Materials. are MP4 files provided as separated files. They showed the R^2 values between the component index and the spectral index calculated from the fluorescence spectrum obtained by scanning the excitation wavelengths as contour maps with the fluorescence wavelength.

Figure. S38. GIF showing the R^2 values between chlorophyll content and NDSI calculated from the fluorescence spectrum obtained by scanning the excitation wavelengths as contour maps with the fluorescence wavelength.

Figure. S39. GIF showing the R^2 values between TPC aNDSI calculated from the fluorescence spectrum obtained by scanning the excitation wavelengths as contour maps with the fluorescence wavelength.

Figure. S40. GIF showing the R^2 values between TFC and NDSI calculated from the fluorescence spectrum obtained by scanning the excitation wavelengths as contour maps with the fluorescence wavelength.

Figure S41. GIF showing the R^2 values between RA content and NDSI calculated from the fluorescence spectrum obtained by scanning the excitation wavelengths as contour maps with the fluorescence wavelength.

Figure S42. GIF showing the R^2 values between DPPH radical scavenging activity and NDSI calculated from the fluorescence spectrum obtained by scanning the excitation wavelengths as contour maps with the fluorescence wavelength.

Figure S43. GIF showing the R^2 values between FRAP and NDSI calculated from the

fluorescence spectrum obtained by scanning the excitation wavelengths as contour maps with the fluorescence wavelength.

Figure S44. GIF showing the R^2 values between ORAC and NDSI calculated from the fluorescence spectrum obtained by scanning the excitation wavelengths as contour maps with the fluorescence wavelength.

Figure S45. GIF showing the R^2 values between Aβ aggregation inhibitory activity and NDSI calculated from the fluorescence spectrum obtained by scanning the excitation wavelengths as contour maps with the fluorescence wavelength.

Figure S46. GIF showing the R^2 values between chlorophyll content and RSI calculated from the fluorescence spectrum obtained by scanning the excitation wavelengths as contour maps with the fluorescence wavelength.

Figure S47. GIF showing the R^2 values between TPC and RSI calculated from the fluorescence spectrum obtained by scanning the excitation wavelengths as contour maps with the fluorescence wavelength.

Figure S48. GIF showing the R^2 values between TFC and RSI calculated from the fluorescence spectrum obtained by scanning the excitation wavelengths as contour maps with the fluorescence wavelength.

Figure S49. GIF showing the R^2 values between RA content and RSI calculated from the fluorescence spectrum obtained by scanning the excitation wavelengths as contour maps with the fluorescence wavelength.

Figure S50. GIF showing the R^2 values between DPPH radical scavenging activity and RSI calculated from the fluorescence spectrum obtained by scanning the excitation wavelengths as contour maps with the fluorescence wavelength.

Figure S51. GIF showing the R^2 values between FRAP and RSI calculated from the fluorescence spectrum obtained by scanning the excitation wavelengths as contour maps with the fluorescence wavelength.

Figure S52. GIF showing the R^2 values between ORAC and RSI calculated from the fluorescence spectrum obtained by scanning the excitation wavelengths as contour maps with the fluorescence wavelength.

Figure S53. GIF showing the R^2 values between Aβ aggregation inhibitory activity and RSI calculated from the fluorescence spectrum obtained by scanning the excitation wavelengths as contour maps with the fluorescence wavelength.

Figure S54. GIF showing the R^2 values between chlorophyll content and DSI calculated from the fluorescence spectrum obtained by scanning the excitation wavelengths as contour maps with the fluorescence wavelength.

Figure S55. GIF showing the R^2 values between TPC and DSI calculated from the fluorescence spectrum obtained by scanning the excitation wavelengths as contour maps with the fluorescence wavelength.

Figure S56. GIF showing the R^2 values between TFC and DSI calculated from the fluorescence spectrum obtained by scanning the excitation wavelengths as contour maps with the

fluorescence wavelength.

Figure S57. GIF showing the R^2 values between RA content and DSI calculated from the fluorescence spectrum obtained by scanning the excitation wavelengths as contour maps with the fluorescence wavelength.

Figure S58. GIF showing the R^2 values between DPPH radical scavenging activity and DSI calculated from the fluorescence spectrum obtained by scanning the excitation wavelengths as contour maps with the fluorescence wavelength.

Figure S59. GIF showing the R^2 values between FRAP and DSI calculated from the fluorescence spectrum obtained by scanning the excitation wavelengths as contour maps with the fluorescence wavelength.

Figure S60. GIF showing the R^2 values between ORAC and DSI calculated from the fluorescence spectrum obtained by scanning the excitation wavelengths as contour maps with the fluorescence wavelength.

Figure S61. GIF showing the R^2 values between Aβ aggregation inhibitory activity and DSI calculated from the fluorescence spectrum obtained by scanning the excitation wavelengths as contour maps with the fluorescence wavelength.

12. Relationship between functionality and ingredient amount

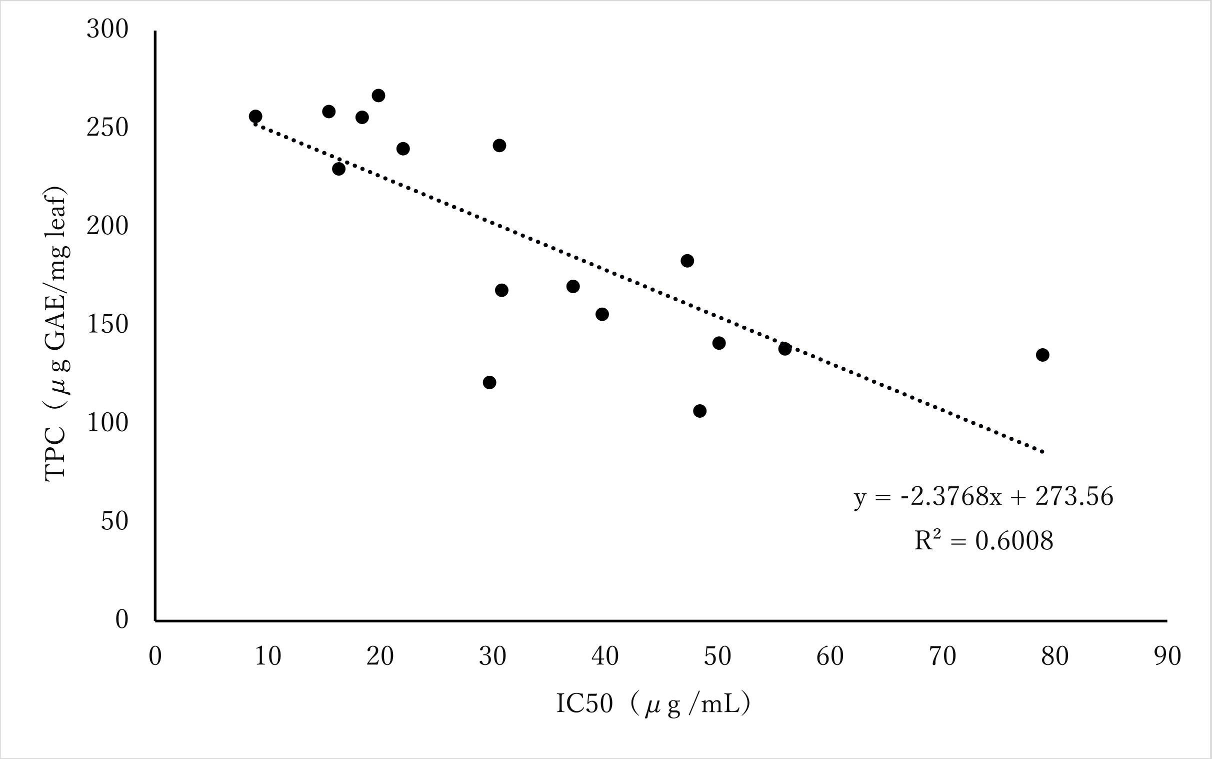

Figure S62 Relationships between DPPH radical scavenging and TPC

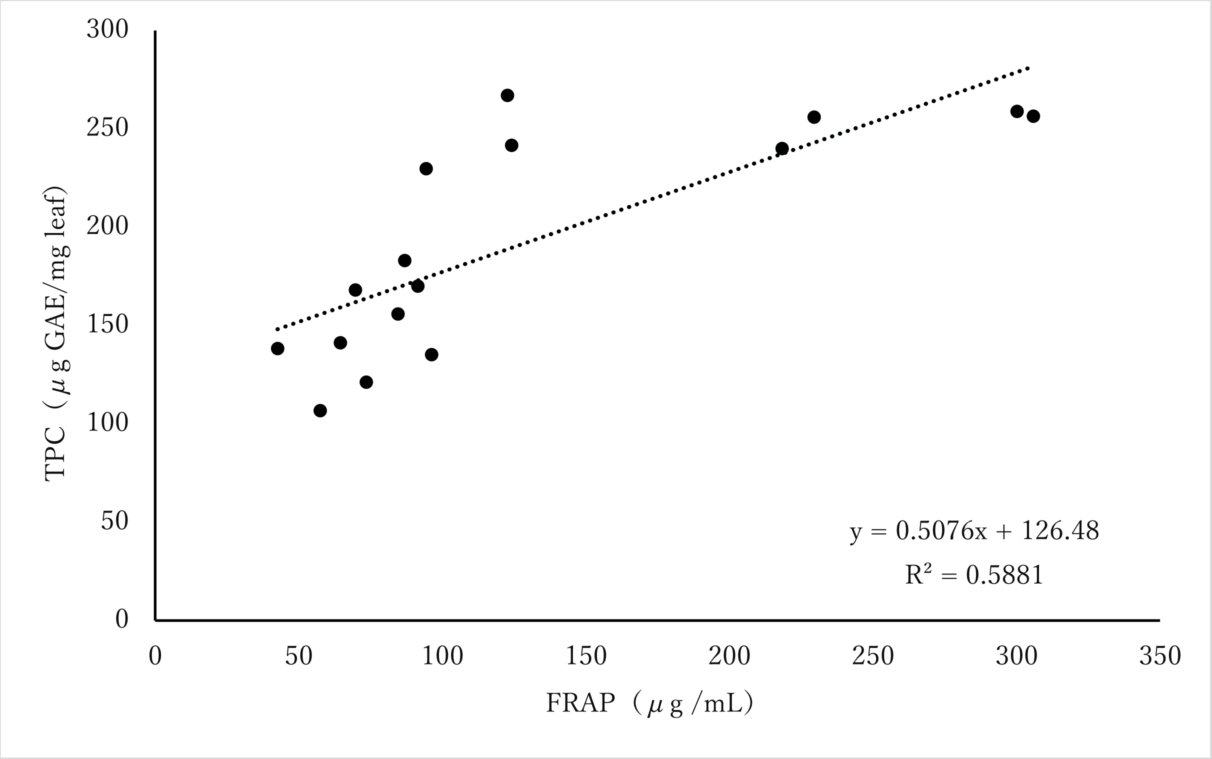

Figure S63 Relationships between FRAP and TPC

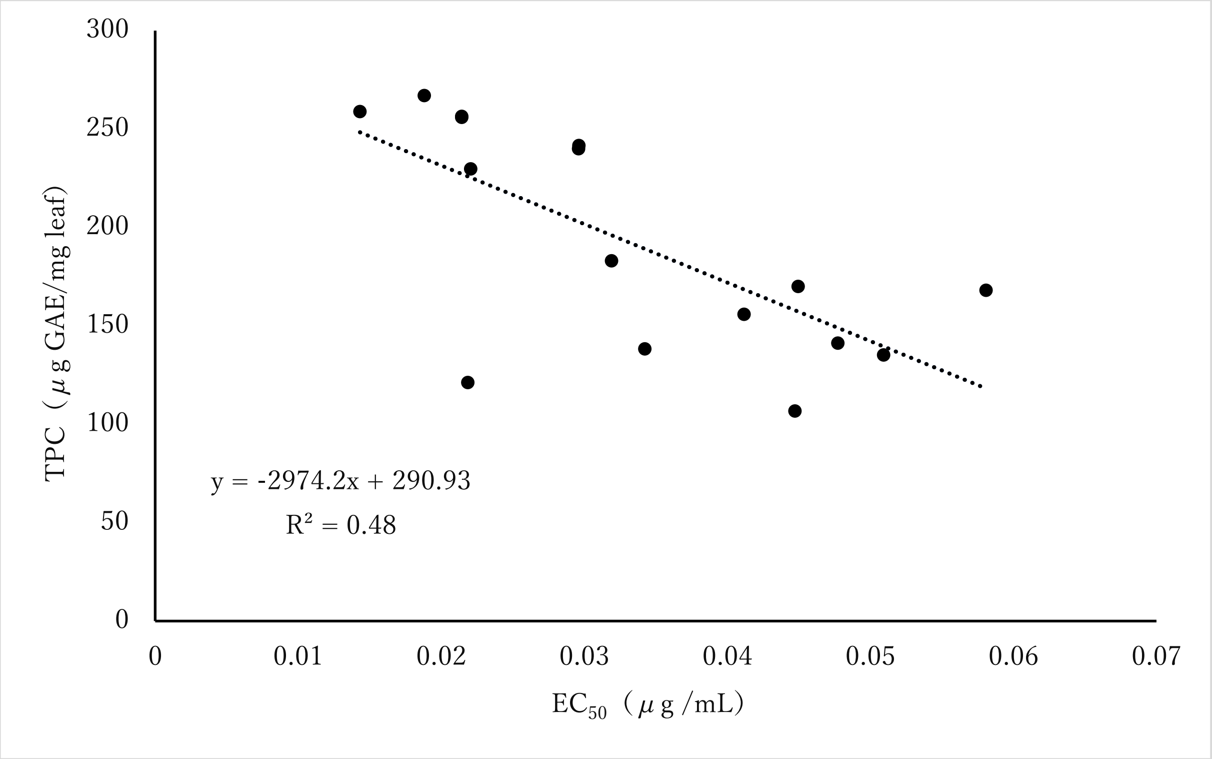

Figure S64 Relationships between Aβ aggregation inhibitory activity and TPC

References

1. Lichtenthaler, H.K. Multi-colour fluorescence imaging of photosyntetic activityand plant stress. Photosynthetica 2021, 59, 364-380.

2. Pora, R.; Thompsonand, W.; Kriedemann, P. Determination of accurate extinction coefficients and simultaneous equations for assaying chlorophylls a and b extracted with four different solvents: verification of the concentration of chlorophyll standards by atomic absorption spectroscopy. *Biochim. Biophys. Acta* **1989,** 975, 384-394.

3. Singleton, V. L.; Orthofer, R.; Lamuela-Raventos, R. M.; Analysis of total phenols and other oxidationn substrates and antioxidants by means of Folin-Ciocalteu reagent. *Polyphenols and Flavonoids* **1999,** 299, 152-178.

4. Ricardo, G. W.; Antonio, S. 1998. Analysis of propolis: some parameters and procedures for chemical quality control. J. Apicult. Res., 37(2), 99-105.

5. Shimada, K.; Fujikawa, K.; Yahara, K.; Nakamura, T. Antioxidative properties of xanthan on the autoxidation of soybean oil in cyclodextrin emulsion. *J. Agric. Food Chem.* **1992,** 40, 945-948.

6. Oyaizu, M. Studies on the product of browning reaction prepared from glucose amine. *Jpn. J. Nutr.* **1986,** 44, 307-315.

7. Dasgupta, S.; Pandya, M.; Patel, N. Study on antioxidant activities of some less utilized edible fruits. *Techno. Innov. Pharm. Res.* **2001**, 5, 24-32.

8. Ou, B.; Hampsch-Woodill, M.; Prior, R. L. Information development and validation of an improved oxygen radical absorbance capacity assay using fluorescein as the fluorescent probe, *J. Agric. Food Chem.* **2001,** 10, 4619-4626.

9. Ishigaki, Y.; Tanaka, H.; Akama, H.; Ogara, T.; Uwai, K.; Tokuraku, K.; A microliter-scale high-throughput screening system with quantum-dot nanoprobes for amyloid-β aggregation inhibitors. *Plos One*. **2013**, 8(8), e72992.

10. Sasaki, R.; Tainaka, R.; Ando, Y.; Hashi, Y.; Deepak, H.V.; Suga, Y.; Murai, Y.; Anetai, M.; Monde, K.; Ohta, K.; et al. An Automated Microliter-Scale High-Throughput Screening System (MSHTS) for Real-Time Monitoring of Protein Aggre-gation Using Quantum-Dot Nanoprobes. Sci. Rep. 2019, 9, 2587.
